# Idiopathic lobular panniculitis of pregnancy with resolution following delivery

**DOI:** 10.1016/j.jdcr.2023.08.031

**Published:** 2023-09-05

**Authors:** Danielle N. Turner, Jose L. Cortez, Elizabeth S. Garchar, Hillary Elwood, Nikifor K. Konstantinov

**Affiliations:** a University of New Mexico, School of Medicine, Albuquerque, New Mexico; b Department of Dermatology, University of New Mexico, Albuquerque, New Mexico; c Department of Obstetrics and Gynecology, University of New Mexico, Albuquerque, New Mexico; d Department of Dermatopathology, TriCore Reference Laboratories, Albuquerque, New Mexico

**Keywords:** idiopathic lobular panniculitis, pregnancy

## Introduction

Idiopathic lobular panniculitis (ILP) is a rare inflammatory disorder of the subcutaneous adipose tissue and usually presents with fevers and may have systemic organ involvement. ILP is not extensively described in the literature. It is a diagnosis of exclusion, and an extensive systemic workup should be performed to rule out other causes. Here we describe a case of skin-limited ILP in a woman during her third trimester of pregnancy, which spontaneously resolved after delivery.

## Case report

A 36-year-old female with a history of morbid obesity and diabetes mellitus without a history of cold exposure or injury presented at 34 weeks’ gestation to the hospital with a 2-week history of pain, swelling, and redness of her right lower extremity which did not improve on outpatient courses of cephalexin and trimethoprim-sulfamethoxazole for suspected cellulitis.

**Fig 1:**
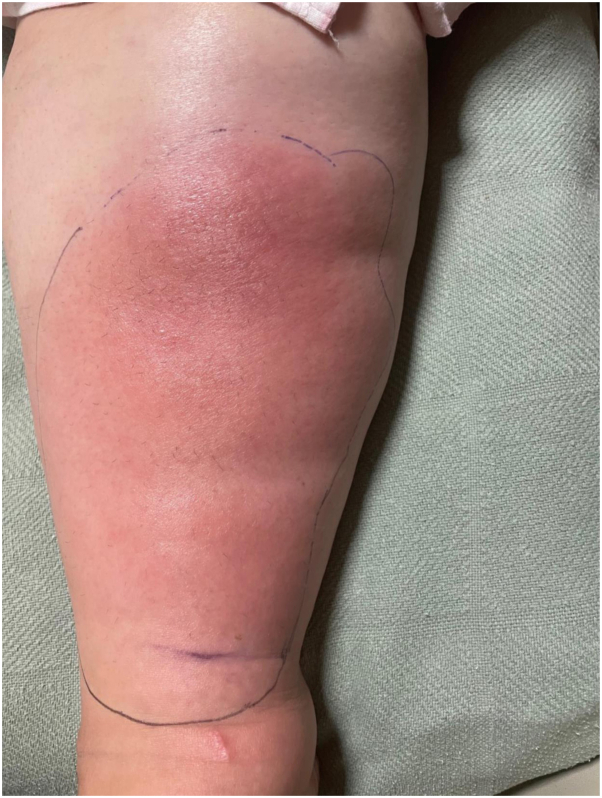
Clinical presentation of idiopathic lobular panniculitis. Large indurated and edematous ill-defined *pink*-to-*red* plaque involving the right shin.

On admission, the patient was afebrile and hemodynamically stable. Physical exam was notable for a large red indurated, edematous plaque on the right shin with tenderness on palpation (Fig 1). Right lower extremity ultrasound demonstrated soft tissue edema without a drainable collection. The patient was started on vancomycin and ceftriaxone. There was no improvement on the broad antibiotics after 1 week and skin biopsies were obtained.

**Fig 2:**
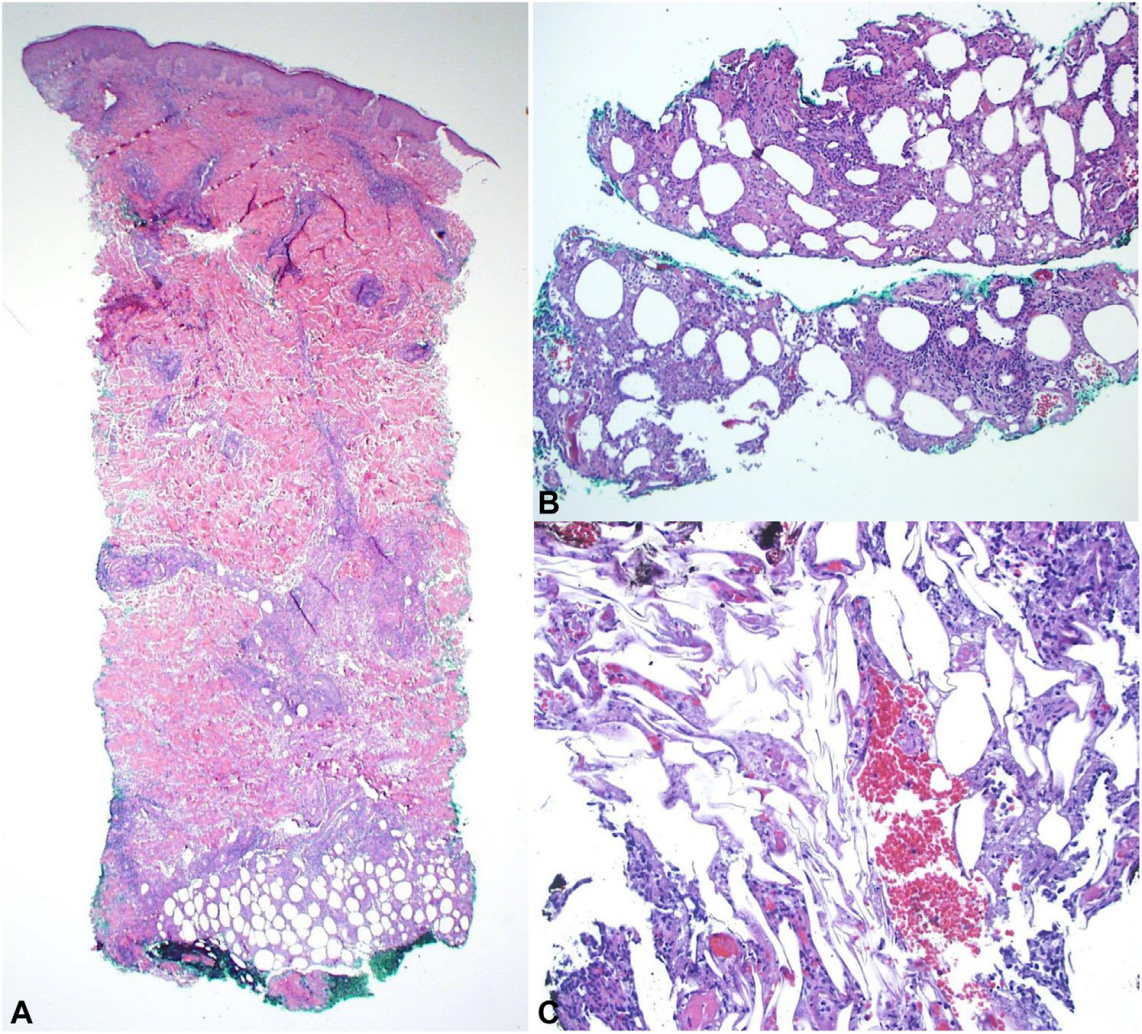
Dermatopathology findings of idiopathic lobular panniculitis. **A,** Sections stained with hematoxylin and eosin demonstrate a lobular infiltrate at the base of the biopsy (magnification 2×). **B,** Separate fragment of subcutis demonstrating lobular infiltrate with necrosis and fibrin thrombi (magnification 10×). **C,** Separate fragment of subcutis demonstrating lobular infiltrate with necrosis and fibrin thrombi (magnification 20×).

A 6 millimeter telescoping punch biopsy of the skin demonstrated a lymphocyte-predominant lobular subcutaneous infiltrate with admixed fibrin thrombi, scattered neutrophils, and fat necrosis (Fig 2). The epidermis was uninvolved and the dermis demonstrated a perivascular and periadnexal lymphohistiocytic infiltrate with negative Alcian blue stain for mucin (Fig 2). The CD3+ subcutaneous lymphocytes did not rim the adipocytes and consist of a mix of CD4+ and CD8+ cells. Tissue cultures of skin for bacterial, mycobacterial, and fungal organisms were negative.

Further workup was only notable for an elevated erythrocyte sedimentation rate to 99 (normal < 38) and C-reactive protein to 6 (normal <0.9). Complete blood count, comprehensive metabolic panel, lactate dehydrogenase, peripheral blood smear and infectious workup (hepatitis B and C serologies, HIV, QuantiFERON gold, coccidiomycosis, histoplasmosis, Coxiella, bartonella, and syphilis screening) were all normal. Antinuclear antibodies, including extractable nuclear antibody panel, double-stranded DNA, antiphospholipid antibody panel, complement levels (C3/C4), cryoglobulins, and cryofibrinogens were all normal.

The patient was started on topical fluocinonide 0.05% ointment and experienced moderate improvement. Following delivery, the patient experienced rapid resolution of her symptoms and discontinued steroids. At 3-month clinic follow-up, she remained asymptomatic with resolved cutaneous lesions.

## Discussion

Panniculitides are categorized as predominantly septal or lobular as indicated by histopathologic studies. The classification of the panniculitides also depends on the location of the inflammatory cell infiltrate, the type of the inflammatory cell infiltrate, the presence or absence of vasculitis, and the presence or absence of necrosis.^1^, ^2^, ^3^ Patients typically develop indurated, edematous, tender plaques, or nodules in areas of adiposity. Anatomic location is generally on the lower extremities although this may vary, depending on the specific type of panniculitis. The presence of panniculitides is often an indication that a patient has an underlying condition.^1^, ^2^, ^3^

Pregnancy is 1 of the common causes of erythema nodosum, the most common panniculitis, which is predominantly a septal panniculitis.^2^ Lobular panniculitis development in pregnancy, however, is rare and extensive evaluation should be taken to rule out lupus panniculitis, subcutaneous panniculitis-like-T-cell lymphoma, and erythema induratum (EI). EI can be due to infections, autoimmune disorders, hypothyroidism, and inflammatory bowel disease.^4^ On histopathology, EI presents as a lobular panniculitis with neutrophilic-predominant vasculitis, which was absent in our patient.^1^ Our case is distinct because histopathology demonstrated a lobular lymphocytic panniculitis with fat necrosis and fibrin thrombi, without vasculitis or rimming of adipocytes. Immunohistochemistry, tissue cultures, and autoimmune serologies were normal, making infectious panniculitis, lupus panniculitis, subcutaneous panniculitis-like-T-cell lymphoma, and EI unlikely, leading to ILP as the most likely diagnosis.

ILP typically presents on the lower extremities with ill-defined tender subcutaneous nodules and swelling. Patients with ILP may have systemic involvement, which is characterized by relapsing fevers and may have systemic organ involvement. The liver is frequently involved and may lead to cirrhosis with hepatomegaly, jaundice, increase in transaminases, and lactate dehydrogenase. Hematologic manifestations are also seen in which a hypochromic, microcytic anemia is seen, as well as thrombocytopenia. Coagulopathies, which may lead to hemorrhagic complications with internal organ bleeding and disseminated intravascular coagulation, are an important cause of patient morbidity and mortality. Renal manifestations, with proteinuria and either membranous or proliferative glomerulonephritis have been described. Perivisceral fat involvement (in the pericardium, pleura, omentum, and mesentery) may also occur.^5^

One other case of ILP in the beginning of the second trimester of pregnancy has been reported, in which the patient presented with high fevers and right thigh and lower extremity panniculitis. The patient in that case was successfully treated with systemic corticosteroids.^6^ Our patient had a skin-limited form of ILP, which developed in her third trimester of pregnancy and resolved with delivery and topical steroids. The development of panniculitides in pregnancy is not completely understood. There are several hypotheses which link the association of pregnancy with panniculitis. Pregnancy state is a known immune modulator and 1 theory is that a type 4 hypersensitivity reaction to progesterone or estrogen plays a role in the development of panniculitis. An additional pathogenetic link between pregnancy and panniculitis may be related to tissue damage caused by the release of inflammatory mediators such as E-selectin, P-selectin, platelet endothelial cell adhesion molecule, vascular cell adhesion molecule-1, and intercellular adhesion molecule-1, in response to activation of the complement system as a result of the deposition of immune complexes within the subcutaneous tissue.^7^^,^^8^

This case has value in recognizing a rare form of panniculitis that may develop during pregnancy which had a benign clinical course after delivery. Despite that ILP is rarely observed, it adds to the list of pregnancy-associated dermatoses that obstetricians and dermatologists need to be aware of, particularly as a mimicker of cellulitis and other causes of panniculitis.

## Conflicts of interest

None disclosed.
